# Supplementary material for: Deformed Mediated Larval Incisor Lobe Development Causes Differing Feeding Behavior between Oriental Armyworm and Fall Armyworm
Source: Insects. 2022 Jun 29;13(7):594. doi: 10.3390/insects13070594 (PMC9320430; doi:10.3390/insects13070594)
Supplement: Supplementary file 1 [file insects-13-00594-s001.zip › insects-1752115-supplementary.pdf]

Table S1. Primers used in this study

No	Name	Sequence (5' > 3')	Function	Gene
1	MsDfdFLS	ATGAGCTCATTCCTCACCAACG	Cloning	MsDfd
2	MsDfdFLA	TTATAAGGCGGTTAGACCGTAGT		
3	SfDfdFLS	ATGAGCTCATTTCTTACCAACG		SfDfd
4	SfDfdFLA	TTATAAGGCGGTTAGACCGTA		
5	MsDfdQS	AGCTCCATGCTTTAGGACGA	RT-qPCR	MsDfd
6	MsDfdQA	TGGTTCCATTCCAGGTTGAT		
7	Msrp49QS	TGACAAACTCAAGCGTAACTGGCG		rp49
8	Msrp49QA	TTGCGGAAACCATTGGGCAG		
9	SfDfdQS	AACCACGACTAAGGGCACAC		SfDfd
10	SfDfdQA	TGTCATAGGATGCCCTGGAT		
11	SfRPL18QS	CGTATCAACCGACCTCCACT		RPL18
12	SfRPL18QA	AGGCACCTTGTAGAGCCTCA		
13	MsDfddsS	TAATACGACTCACTATAGGGAACTGCCCAACACCAAAAAC	RNAi	MsDfd
14	MsDfddsA	TAATACGACTCACTATAGGGGGCGGTTAGACCGTAGTCTG		
15	SfDfddsS	TAATACGACTCACTATAGGGCAGCCCATAACCCTGACACT		SfDfd
16	SfDfddsA	TAATACGACTCACTATAGGGGGTTCCATTCCAGGTTGAAA		
17	GFPdsS	TAATACGACTCACTATAGGGAGTGCTTCAGCCGCTACCC		GFP
18	GFPdsA	TAATACGACTCACTATAGGGGCGCTTCTCGTTGGGGTC		

Table S2. Dfds used for the phylogenetic tree construction.

No.	Genes	Species	Accession number
1	Hox-B4	*Papilio xuthus*	>XP_013173802.1
2	Hox-B4	*Papilio machaon*	>XP_014359268.1
3	Hox-B4	*Papilio polytes*	>XP_013139547.1
4	Hox-B4	*Amyelois transitella*	>XP_013193972.1
5	deformed	*Bombyx mori*	>NP_001037341.1
6	Hox-C4	*Manduca sexta*	>XP_037297049.1
7	deformed	*Galleria mellonella*	>XP_026759529.1
8	Hox-C4	*Zerene cesonia*	>XP_038221672.1
9	Hox-B4	*Pieris rapae*	>XP_022113566.1
10	Hox-B4-like	*Aricia agestis*	>XP_041968511.1
11	Hox-B4-like	*Ostrinia furnacalis*	>XP_028170681.1
12	hypothetical protein	*Chilo suppressalis*	>RVE51778.1
13	Hox-B4	*Trichoplusia ni*	>XP_026731728.1
14	Hox-C4	*Plutella xylostella*	>XP_037976115.1
15	deformed	*Spodoptera exigua*	>AYI99245.1
16	Hox-B4	*Spodoptera litura*	>XP_022826302.1
17	Hox-B4	*Helicoverpa armigera*	>XP_021196577.1
18	deformed	*Culex quinquefasciatus*	>XP_038105254.1
19	deformed-like	*Ctenocephalides felis*	>XP_026471792.1
20	Hox-C4a	*Onthophagus taurus*	>XP_022907652.1
21	Hox-C4-like	*Photinus pyralis*	>XP_031341519.1
22	Hox-C4	*Sitophilus oryzae*	>XP_030758210.1
23	Hox-C4	*Aethina tumida*	>XP_019868479.1

24	Hox-C4-like	*Leptinotarsa decemlineata*	>XP_023012021.1
25	Hox-C4	*Anoplophora glabripennis*	>XP_018562493.1
26	Hox-C4	*Tribolium madens*	>XP_044259692.1
27	deformed	*Tribolium castaneum*	>NP_001034510.1
